# Spontaneous Uterine Perforation of Pyometra Presenting as Acute Abdomen

**DOI:** 10.1155/2014/738568

**Published:** 2014-06-24

**Authors:** Toshihiro Kitai, Kentaro Okuno, Hiromi Ugaki, Yoshiko Komoto, Satoshi Fujimi, Masahiko Takemura

**Affiliations:** ^1^Departments of Obstetrics and Gynecology, Osaka General Medical Center, 3-1-56 Bandaihigashi, Sumiyoshi-ku, Osaka 558-0056, Japan; ^2^Departments of Emergency and Critical Care, Osaka General Medical Center, 3-1-56 Bandaihigashi, Sumiyoshi-ku, Osaka 558-0056, Japan

## Abstract

Pyometra is the accumulation of pus in the uterine cavity, and spontaneous perforation of pyometra resulting in generalized diffuse peritonitis is extremely uncommon. We report a rare case of diffuse peritonitis caused by spontaneous perforation of pyometra. A 66-year-old postmenopausal woman with diffuse abdominal pain and vomiting was admitted to our institution. She had a history of mixed connective-tissue disease and had been taking steroids for 20 years. Under a diagnosis of generalized peritonitis secondary to perforation of the gastrointestinal tract or uterus, supravaginal hysterectomy and bilateral salpingo-oophorectomy were performed. Unfortunately, wound dehiscence and infection occurred during the postoperative course, which were exacerbated by her immunocompromised state. Despite intensive care and a course of antibiotics, the patient died of multiple organ failure resulting from sepsis on the 36th postoperative day. Although correct diagnosis, early intervention, and proper treatment can reduce morbidity and mortality of spontaneous perforation of pyometra, if severe infection occurs, this disease can be life threatening for immunocompromised hosts.

## 1. Introduction

Pyometra is the accumulation of pus in the uterine cavity resulting from interference with its natural drainage [1], which may or may not be associated with malignancy. As an uncommon condition, it has been reported to occur in 0.1-0.2% of all gynecologic patients and 13.6% of elderly gynecologic outpatients [2]. Pyometra develops gradually and, as it progresses, may enlarge the uterus, causing degenerative changes that may rarely lead to sloughing of the uterine wall with subsequent spillage of contents into the abdominal cavity [3]. Spontaneous perforation of pyometra resulting in generalized diffuse peritonitis is very rare. To date, about 50 case reports of spontaneous perforation of pyometra have been documented in the English literature. This paper reports an additional case of spontaneous uterine perforation of pyometra.

## 2. Case Presentation

**Figure 1:**
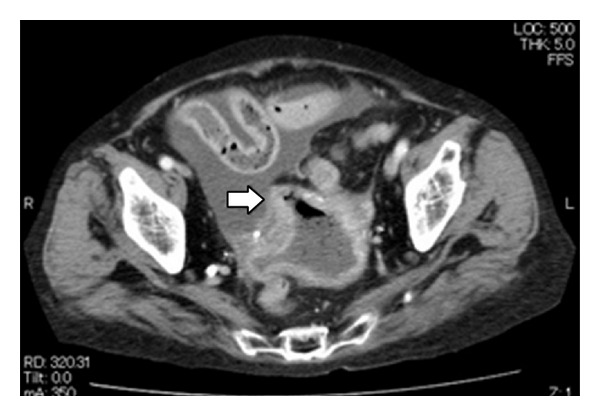
Transverse view of contrast-enhanced computed tomography scan showing the presence of fluid within the abdominal cavity and a significantly distended fluid-filled uterus. In addition, free air was detected in the abdominal and uterine cavities. White arrow indicates the perforation site.

A 66-year-old multiparous postmenopausal woman was admitted because of diffuse abdominal pain and vomiting during the previous 24 hours. She had history of mixed connective-tissue disease and had been taking steroids for 20 years. In addition, she had received hemodialysis for chronic renal failure dating from 2 months previously. Her gynecologic history was unremarkable, and there was no history of postmenopausal bleeding or vaginal discharge. On physical examination, she appeared ill, with vital signs as follows: body temperature 37.3°C, blood pressure 96/61 mmHg, pulse rate 82 beats/min, and oxygen saturation 96% (oxygen mask 4 L/min). Her abdomen was distended, with tenderness in the lower portion. Vaginal examination showed no cervical and vaginal anomalies, vaginal discharge, or detectable pelvic mass. Results of laboratory investigations on admission were as follows: white blood cell (WBC) count 3.0 × 10^3^/*μ*L, hemoglobin 11.3 g/dL, and C-reactive protein (CRP) 7.32 mg/dL. Progression to sepsis was suspected because the WBC count documented at the previous institution was 13.0 × 10^3^/*μ*L, a drastic decline. A contrast-enhanced computed tomography (CT) scan showed the presence of fluid within the abdominal cavity and a significantly distended fluid-filled uterus. In addition, free air was detected in the abdominal and uterine cavities (Figure 1).

**Figure 2:**
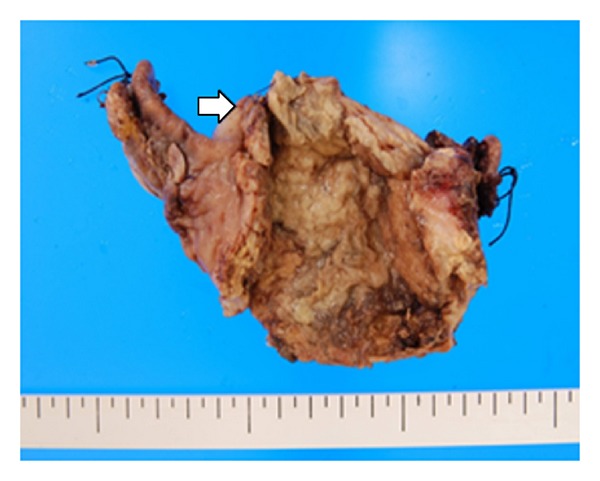
The resected corpus of the uterus. White arrow indicates the perforation site.

A preoperative diagnosis of generalized peritonitis secondary to perforation of the gastrointestinal tract or uterus was established. Consequently, emergent laparotomy was performed. At laparotomy about 300 mL of seropurulent, fluid was encountered in the peritoneal cavity. No abnormal findings were found in the gastrointestinal tract; however, a perforation with a diameter of 1 cm was found in the uterine fundus. The fallopian tubes and ovaries were normal. A supravaginal hysterectomy and bilateral salpingo-oophorectomy were performed (Figure 2).

Culture of the pus grew* Klebsiella pneumoniae*,* Escherichia coli*, and* Bacteroides fragilis*. Histologic examination revealed pyometra with no evidence of malignancy. Cervical cytology performed after surgery was negative for intraepithelial lesion or malignancy.

**Figure 3:**
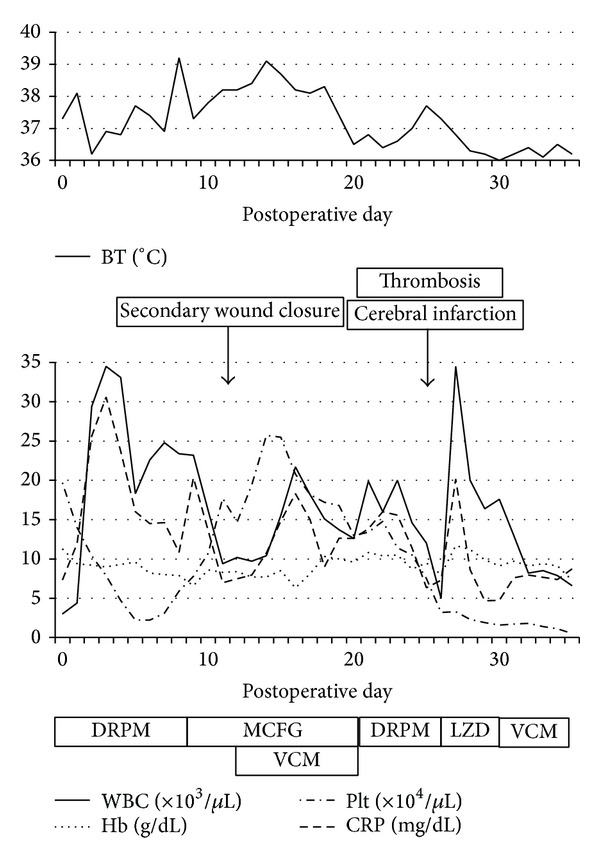
Postoperative course. BT: body temperature; WBC: white blood cell count; Hb: hemoglobin; Plt: platelet; CRP: C-reactive protein; DRPM: doripenem; MCFG: micafungin; VCM: vancomycin; LZD: linezolid.

Figure 3 shows the postoperative course. The patient was admitted to the intensive care unit with strict management of respiration and circulation. Under antibiotic therapy with doripenem (750 mg/day, daily administration), her condition improved over time and she was transferred to the gynecology unit on postoperative day (POD) 8. However, she broke into high fever on POD 9. Antibiotics were changed from doripenem to micafungin (75 mg/day, daily administration) because the culture of her sputum grew* Candida albicans* and *β*-d-glucan was increased in the blood. Subsequently, wound dehiscence occurred, and secondary wound closure was performed on POD 11. She was transferred to the intensive care unit again after surgery, and vancomycin (500 mg/day, alternate-day administration) was added to antibiotic therapy because methicillin-resistant* Staphylococcus aureus* was detected from wound culture. From POD 11, high fever continued and platelet count declined once again. Although hepatic and renal dysfunctions were not revealed, sepsis and multiple organ failure were suspected. Despite the readministration of doripenem, thromboses of the inferior vena cava caused by infection and cerebral infarction were found on POD 26. Although antibiotics were changed from doripenem to linezolid (600 mg/day, daily administration) or vancomycin, sepsis grew progressively worse. Despite exhaustive clinical efforts, the patient died on POD 36.

## 3. Discussion

Pyometra is rare in the general population but more common in elderly women. It is caused as a result of occlusion of the cervical canal by malignant or benign tumors, surgery, radiotherapy, or senile cervicitis [11]. Pyometra is a difficult diagnosis. The classic triad of symptoms is postmenopausal bleeding, vaginal discharge, and lower abdominal pain, but none of these is specific for pyometra and some patients have no symptoms at all [29]. Spontaneous perforation of pyometra is no less rare. To date, only 50 case reports have been documented in the English literature.

A PubMed literature search using key words such as “pyometra,” “perforation,” and “rupture” was performed, and only English-language articles were collected.  Table 1 summarizes the 42 cases of spontaneous perforation of pyometra during the past decade, from 2004 to 2013, including our case. The median age was 75 years (range, 40–93). The common clinical symptoms were abdominal pain (41 cases, 97.6%), fever (23 cases, 54.8%), and vomiting (13 cases, 31.0%). No case had genital bleeding and five cases (11.9%) were in shock at the time of admission. Data regarding the associated malignancy were available in all 42 cases. Of the 11 cases (26.2%) with malignant tumor, eight (72.7%) had cervical cancer and three (27.3%) had sigmoid colon cancer. Although preoperative diagnosis was confirmed in all 42 cases, an accurate preoperative diagnosis was made in only 13 cases (30.9%). The other preoperative diagnoses included perforation of the gastrointestinal tract in 20 cases (47.6%), generalized peritonitis in five cases (11.9%), appendicitis in one case (2.4%), pneumoperitoneum in one case (2.4%), mesenteric artery ischemia in one case (2.4%), and incarcerated hernia in one case (2.4%). The low preoperative diagnostic rate suggested that preoperative diagnosis of spontaneous perforation of pyometra was difficult. Laparotomy was performed in all 42 cases as an initial treatment. Total, subtotal, or supravaginal hysterectomies were performed in 37 cases (88.1%), drainage in four cases (9.5%), and surgical closure of perforated uterine wall in one case (2.4%). Data regarding the perforation site were available in 35 cases. The sites of uterine perforation were the fundus in 27 cases (77.1%), anterior in five cases (14.3%), and posterior in three cases (8.6%). Bacteriological studies of peritoneal pus were performed in 25 cases. The common etiological organisms were* Escherichia coli* (10 cases, 40.0%) and* Bacteroides* species (five cases, 20.0%). The other bacteria were* Klebsiella*,* Streptococcus*,* Staphylococcus*,* Acinetobacter*,* Porphyromonas*,* Enterococcus*, and* Actinomyces* species. No bacteria were detected from the culture in four cases (16.0%). Data regarding complicated diseases were available in 29 cases. Nine cases (31.0%) involved an immunocompromised state, such as renal failure, diabetes mellitus, and steroid administration. The prognoses were documented in 33 cases. The total number of patients who died was five (15.2%). Of those in an immunocompromised state, four patients died, or the mortality rate was 44.4%. This may suggest that immunocompromised hosts represent a group at high risk for spontaneous perforation of pyometra. It is hoped that further cases are accumulated and that statistical analysis is performed.

## 4. Conclusion

Although correct diagnosis, early intervention, and proper treatment can reduce morbidity and mortality of spontaneous perforation of pyometra, if severe infection occurs, this disease can be life threatening for immunocompromised hosts.

## Figures and Tables

**Table 1:** Cases of spontaneous perforation of pyometra from the literature review and our own case.

Patients	First author	Age	Symptoms	Preoperative diagnosis	Associated malignancy	Perforation site	Bacterial culture	Treatment	Immunocompromised state	Outcome
1	Nuamah [4]	79	AP, F, and V	PGIT	None	Fundus	ND	TAH + BSO	ND	Died (MOF)
2	Shahid [5]	80	AP	PGIT	CC	Fundus	ND	TAH + BSO	ND	Survived
3	Chan [6]	73	AP, shock	PP	None	Fundus	*Klebsiella pneumoniae*, *Streptococcus viridans *	TAH + BSO	DM	Survived
4	Tsai [7]	40	AP, F	GP	None	Fundus	ND	STH	ND	Survived
5	Yildizhan [8]	92	AP, V	PGIT	None	Fundus	*Escherichia coli*, *Bacteroides fragilis *	TAH + BSO	ND	Survived
6	Geranpayeh [9]	63	AP, F, and V	PGIT	None	ND	Negative	TAH + BSO	ND	Survived
7	Lee [10]	60	AP, F	PGIT	CC	Fundus	*B. fragilis *	TAH + BSO	DM, RF	ND
8	Saha [3]	60	AP	PGIT	None	Fundus	*Staphylococcus aureus *	SVH + BSO	ND	Survived
9	Li [11]	69	AP, F, and V	PGIT, GP	None	Anterior	*B. fragilis *	SVH + BSO	None	Survived
10	Vyas [12]	60	AP, V	PP	CC	Fundus	*Acinetobacter* species	Drainage	ND	Survived
11	Izumi [13]	83	AP	PP	None	Fundus	*Bacteroides distasonis*, *Porphyromonas asaccharolytica*, and *Streptococcus oralis *	TAH	None	Survived
12	Ou [14]	78	AP, F	PGIT	SCC	ND	ND	TAH + BSO	ND	ND
13		54	AP, F	Pneumoperitoneum	None	ND	ND	TAH + BSO	ND	ND
14		78	AP	PP	CC	ND	ND	TAH + BSO	ND	ND
15		80	AP	PGIT	CC	ND	ND	Drainage	ND	ND
16		73	AP, F	PP	CC	ND	ND	TAH + BSO	ND	ND
17		81	AP, F	GP	None	ND	ND	TAH	ND	ND
18	Kim [15]	80	AP	PP	None	Fundus	ND	TAH + BSO	None	Survived
19	Chen [16]	68	AP, F	PGIT	SCC	Fundus	ND	TAH + BSO + OME	ND	Survived
20	Agarwal [17]	60	AP, F	PGIT	CC	Fundus	ND	Drainage	ND	Survived
21	Stunell [18]	64	AP	PP	None	Fundus	ND	TAH + BSO	None	ND
22	Lim [19]	89	AP, F	GPIT	None	Fundus	*E. coli *	TAH + BSO	None	Survived
23		87	AP	GPIT	None	Fundus	*Enterococcus faecalis *	Surgical closure	None	ND
24	Chaopotong [20]	88	AP, F, and V	GP	None	Fundus	Negative	TAH + BSO	DM	Died (sepsis)
25	Sahoo [21]	50	AP, F, and shock	GPIT	None	Fundus	ND	TAH + BSO	ND	Survived
26	Shapey [22]	84	AP, V	GPIT	None	Anterior	Negative	TAH	Steroid administration	Survived
27	Ikeda [23]	80	AP	PP	CC	Anterior	*E. coli *	TAH + BSO	None	Survived
28		81	AP, F	PGIT	None	Fundus	*E. coli *	TAH + BSO	None	Survived
29		93	AP, F	PGIT	None	Posterior	*K. pneumoniae*, *Enterococcus faecalis*, and *Streptococcus* species	TAH	None	Survived
30		84	AP	PGIT	SCC	Anterior	*E. coli *	TAH + BSO	None	Survived
31		74	AP, F	PP	None	Fundus	*E. coli*, *Staphylococcus epidermidis *	STH + BSO	None	Survived
32		79	AP, F	Appendicitis	None	Fundus	*E. coli *	TAH	None	Survived
33		66	Shock	PP	None	Anterior	*K. pneumoniae *	Drainage	DM	Died (strangulation ileus)
34	Kutuk [24]	71	AP, F, and V	GP	None	Fundus	*E. coli *	TAH + BSO	ND	Survived
35		75	AP, V	Mesenteric artery ischemia	None	Fundus	Negative	TAH + BSO	None	Survived
36		68	AP	GPIT	None	Posterior	*E. coli *	TAH + BSO	RF	Died (sepsis)
37	Hagiya [25]	86	AP, shock	PP	None	Fundus	*Actinomyces* species	TAH + BSO	DM	Survived
38	Mallah [26]	78	AP, F, and V	Incarcerated hernia	None	Fundus	ND	TAH + BSO	None	Survived
39		61	AP, F, V, and shock	GP	None	Posterior	ND	TAH + BSO	None	Survived
40	Abu-Zaid [27]	63	AP, F, and V	PP	None	Fundus	*Streptococcus constellatus *	TAH + BSO	None	Survived
41	Patil [28]	74	AP, F	PGIT	None	Fundus	ND	TAH + BSO	DM	Survived
42	Kitai [present case]	66	AP, V	PP	None	Fundus	*E. coli*, *B. fragilis *	SVH + BSO	RF, steroid administration	Died (sepsis)

AP: abdominal pain; F: fever; V: vomiting; PGIT: perforation of the gastrointestinal tract; PP: perforation of pyometra; GP: generalized peritonitis; CC: cervical cancer; SCC: sigmoid colon cancer; TAH: total abdominal hysterectomy; STH: subtotal hysterectomy; SVH: supravaginal hysterectomy; BSO: bilateral salpingo-oophorectomy; DM: diabetes mellitus; RF: renal failure; MOF: multiple organ failure.
